# Recurrent Mesonephric-like Adenocarcinoma of the Ovary with Bladder and Vaginal Recurrences: A Diagnostic Challenge

**DOI:** 10.3390/diagnostics16193099

**Published:** 2026-09-24

**Authors:** Anca Evsei-Seceleanu, Tudor Turcu, Aurelian Mihai Ghita, Sandu-Dumitru Ramboiu, Alexandru Ciobanu, Patricia Visan

**Affiliations:** 1 Faculty of Medicine, Carol Davila University of Medicine and Pharmacy, 050474 Bucharest, Romania; 2 Department of Pathology, Saint Mary Clinical Hospital, 011192 Bucharest, Romania; 3 Department of Pathology and Molecular Biology, Pathoteam Diagnostic, 051923 Bucharest, Romania; 4 Department of Surgery I, Clinical County Hospital, 200642 Craiova, Romania; 5 Faculty of Medicine, University of Medicine and Pharmacy, 200349 Craiova, Romania; 6 Department of Radiology, SPAD Imaging, 200352 Craiova, Romania; 7 Medical Oncology Department, OncoClinic Consult, 200094 Craiova, Romania

**Keywords:** mesonephric-like adenocarcinoma, recurrence, ovary, KRAS mutation, case report

## Abstract

**Background and Clinical Significance:** Mesonephric-like adenocarcinoma (MLA) of the ovary is a very rare entity that has only recently been recognized as a distinct subtype in the World Health Organization (WHO) classification. It is an aggressive tumor with a propensity for recurrence. The aim of this case report was to highlight the diagnostic challenges associated with MLA and the importance of immunohistochemical and molecular testing. **Case Presentation:** We report the case of a 59-year-old woman who presented with a left ovarian tumor. Histopathological examination led to an initial diagnosis of low-grade serous carcinoma, and the patient was treated accordingly. Five years later, the tumor recurred in the bladder wall and was initially interpreted as urothelial carcinoma. One year later, a further recurrence was identified at the vaginal cuff. Integrated review of the original ovarian tumor and recurrent lesions, together with immunohistochemical and molecular findings, supported the diagnosis of ovarian MLA with bladder and vaginal recurrences. **Conclusions:** Ovarian MLA is extremely rare and can mimic more common ovarian carcinoma subtypes. Awareness of its morphological and immunohistochemical features is essential to avoid misdiagnosis. MLA should be considered in recurrent gynecologic tumors with mesonephric morphology/immunophenotype even when the recurrence occurs in an unusual site such as the bladder and mimics a primary urinary malignancy.

## 1. Introduction

Mesonephric-like adenocarcinoma (MLA) of the ovary is an extremely rare and aggressive subtype of ovarian malignancy, with only a limited number of cases reported in the literature [1] Mesonephric-like adenocarcinoma is more commonly recognized in the uterine corpus, whereas true mesonephric adenocarcinoma most commonly arises in the cervix. Ovarian MLA was recognized as a distinct tumor entity in the fifth edition of the WHO Classification of Female Genital Tumors in 2020 [2].

The pathogenesis of ovarian MLA remains incompletely understood. It has been hypothesized that these tumors arise from Müllerian carcinomas that subsequently develop mesonephric differentiation. This hypothesis is supported by the frequent association of MLA with endometriosis, serous borderline tumors, or low-grade serous carcinoma, as well as by shared molecular features [3].

The present case is particularly instructive because the primary ovarian tumor and two anatomically distinct recurrences received different initial diagnoses, illustrating the diagnostic pitfalls of MLA across both gynecologic and urinary tract specimens.

## 2. Case Presentation

In 2020, a 59-year-old woman with fatigue, abdominal discomfort, diarrhea, constipation, and weight loss presented to the emergency department with colicky abdominal pain. She was referred to the surgical department for further evaluation. Computed tomography (CT) revealed a left ovarian tumor measuring 82 × 94 × 52 mm and ascites. Intraoperatively, approximately 1000 mL of fluid was identified and aspirated; cytological examination of the ascites was negative for malignant cells. In the pelvic region, the left ovary was cystic and ruptured, measuring 15 × 10 cm, extending into the intrauterine and rectal spaces and adherent to the base of the mesentery. The patient underwent hysterectomy with bilateral salpingo-oophorectomy and bilateral lymphadenectomy.

Macroscopically, the left ovarian tumor was predominantly cystic, with solid yellowish and mucinous areas. No exophytic papillary projections were identified, and the serosa was ruptured (Figure 1).

The final histopathological diagnosis was low-grade serous carcinoma with an associated serous borderline tumor. No immunohistochemical or molecular testing was performed. The final stage was FIGO IC2. The patient received six cycles of chemotherapy with paclitaxel (175 mg/m^2^, day 1) plus carboplatin (AUC 5, day 1) at 21-day intervals.

In February 2025, CT revealed a nodular lesion at the lateral wall of the bladder, measuring 29 × 20 mm and protruding into the bladder lumen (Figure 2).

Cystoscopy revealed three small tumors, which were excised and submitted for pathological examination. Microscopic examination showed a high-grade adenocarcinoma. Immunohistochemical findings suggested urothelial carcinoma with squamous and glandular differentiation. The tumor cells were positive for GATA3 and CK7 and negative for uroplakin III (Figure 3).

Although the initial histopathological findings suggested urothelial carcinoma, the clinical and oncology teams also considered the possibility that the bladder lesion represented recurrence of the previously treated ovarian carcinoma. Given this diagnostic uncertainty, the oncology team selected a regimen with activity in both urothelial and recurrent ovarian carcinoma. Accordingly, the patient received six cycles of chemotherapy with gemcitabine (1000 mg/m^2^, days 1 and 8) plus carboplatin (AUC 5, day 1) at 21-day intervals.

In October 2025, the patient underwent laparoscopic partial cystectomy. Histopathological examination with immunohistochemical testing was performed at a different laboratory and suggested a carcinoma of gynecological origin, most likely endometrioid carcinoma. According to the pathology report, the tumor cells were found mostly on the bladder serosa and invaded the bladder wall through the detrusor muscle, ulcerating the bladder mucosa. The tumor cells were positive for PAX8, vimentin and p16 and negative for ER, HNF1beta, WT1 and GATA3. P53 was “wild-type”. Pathology slides from this examination were not available.

In March 2026, the patient presented to the emergency department with vaginal bleeding and foul-smelling discharge. Magnetic resonance imaging (MRI) showed thickening of the vaginal cuff, raising suspicion of local tumor recurrence. A biopsy was performed, and microscopic examination revealed a poorly differentiated carcinoma (Figure 4).

Immunohistochemical testing was performed. The tumor cells were positive for CK7, PAX8, GATA3, and TTF1 and negative for uroplakin III, CK20, WT1, and ER (Figure 5). We excluded a urothelial carcinoma and other gynecological carcinomas, such as low-grade serous carcinoma, endometrioid carcinoma, clear cell carcinoma or high-grade carcinoma (Table 1). The final diagnosis was mesonephric-like adenocarcinoma.

Given the inconsistencies among the pathological diagnoses, we decided to reassess the primary ovarian tumor using immunohistochemistry and to perform molecular testing. Hematoxylin and eosin examination showed diverse architectural patterns. The most frequent pattern consisted of small tubules with ductal or glandular morphology; solid areas, clear cell features, and papillary areas were also observed. The tumor cells were cuboidal to columnar, with moderate cytological atypia and amphophilic to eosinophilic cytoplasm. The nuclei showed vesicular chromatin and nuclear grooves (Figure 6).

Immunohistochemically, the tumor cells showed positive staining for TTF1, GATA3, Napsin A, and CD10, with negative ER expression (Figure 7 and Figure 8). p53 showed a wild-type pattern. This immunophenotypic profile, particularly the combined expression of TTF1 and GATA3 with ER negativity and CD10 positivity, supports the diagnosis of MLA when interpreted in the appropriate morphological context. Although none of these markers is individually specific for MLA, their combined expression pattern helps distinguish MLA from its main morphological mimics, including low-grade serous carcinoma and endometrioid carcinoma, which are TTF1 negative, GATA3 negative, ER positive, CD10 negative, as well as urothelial (TTF1 negative, GATA 3 positive, Napsin A negative, CD10 negative) and clear cell carcinomas (TTF1 negative, Napsin A positive, CD10 negative, ER negative)—Table 1. In particular, Napsin A positivity may overlap with clear cell carcinoma and should therefore be interpreted as part of the overall morphological and immunohistochemical profile rather than as an isolated diagnostic marker.

The morphological and immunohistochemical findings across the primary ovarian tumor and subsequent bladder and vaginal recurrences including differential diagnoses are summarized in Table 1.

Molecular profiling was performed on tumor tissue from the vaginal cuff recurrence using the OncoDeep assay, which evaluates mutations in 638 genes, fusions involving 22 driver genes, and genomic signatures including homologous recombination deficiency (HRD), microsatellite instability (MSI), and tumor mutational burden (TMB). NGS was not performed on the primary ovarian tumor or the bladder wall recurrence. Molecular profiling identified a pathogenic KRAS p.G12D (NM_033360.4:c.35G>A) mutation with a variant allele frequency (VAF) of 46.47%, together with a likely pathogenic truncating PTEN p.C211Lfs*32 (NM_000314.8:c.631dup) alteration (VAF 81.68%) and a likely pathogenic frameshift CTNNA1 p.A127Efs*19 (NM_001903.5:c.363_379dup) alteration (VAF 12.59%). Loss-of-heterozygosity (LOH) events involving BRCA1 and BLM were also detected; however, no pathogenic or likely pathogenic single-nucleotide variants (SNVs), insertions/deletions (indels), or copy-number variants (CNVs) were identified in BRCA1 or BRCA2. Genomic signature analysis showed a homologous recombination-proficient phenotype, with a homologous recombination deficiency (HRD) score of 18.2, below the positivity threshold of 37. The tumor was microsatellite stable (MSI, 5.46%) and had a low tumor mutational burden (TMB, 5.56 mutations/Mb). The KRAS p.G12D alteration identified in our case is consistent with the molecular spectrum reported in MLA; however, this finding is not specific to MLA and should therefore be interpreted in conjunction with the characteristic morphological and immunohistochemical features. The biological and potential clinical significance of the concomitant PTEN and CTNNA1 alterations in MLA remains uncertain.

This testing has its limitations. NGS was performed only on the vaginal-cuff recurrence, not on the original ovarian tumor or bladder recurrence. Therefore, we cannot demonstrate molecular clonality between the three lesions. Nevertheless, clinical history, anatomical distribution, morphological features, and immunophenotypic findings support their interpretation as recurrences of the ovarian MLA.

The case was reviewed comprehensively by integrating the available morphological and immunohistochemical findings from the primary ovarian tumor, the initial bladder biopsy, the partial cystectomy specimen, and the vaginal cuff recurrence. Regarding the bladder lesion, the initial biopsy showed a high-grade adenocarcinoma with pseudoglandular architecture and clear-cell features, overlapping with morphological components identified in the primary ovarian tumor, which displayed predominantly pseudoglandular/ductal architecture with focal papillary, solid, and clear-cell areas. Importantly, the overlying urothelium in the bladder biopsy was unaffected. Although GATA3 positivity initially raised the possibility of urothelial carcinoma, the tumor was CK7 positive, CK20 negative, and uroplakin III negative. In the subsequent partial cystectomy specimen, PAX8 positivity supported Müllerian/gynecological differentiation, whereas ER and WT1 were negative. In addition, the bladder lesion showed an extrinsic pattern of involvement, with tumor invasion of the bladder wall from the outside and ulceration of the overlying urothelium. This pattern of growth, together with the morphological similarities to the ovarian tumor and the immunohistochemical findings supporting a gynecological origin, favored secondary bladder involvement by the ovarian tumor rather than an independent primary urothelial carcinoma. We acknowledge that the histological slides from the partial cystectomy were unavailable for our review and that only the original pathology report could be assessed. Consequently, we could not independently reassess the reported GATA3 negativity in this specimen or explain its discrepancy with the GATA3-positive initial bladder biopsy.

The vaginal cuff recurrence showed solid, pseudoglandular, clear-cell, and hobnail morphology, again overlapping with the architectural and cytological features of the primary ovarian tumor. Immunohistochemically, the vaginal lesion was PAX8, TTF1, GATA3, and CK7 positive and ER, WT1, CK20, and uroplakin III negative, with a wild-type p53 staining pattern. This phenotype was concordant with the mesonephric-like differentiation demonstrated in the primary ovarian tumor, which showed TTF1 and GATA3 positivity, luminal membranous CD10 expression, Napsin A positivity, ER negativity, and a wild-type p53 pattern. The combination of the patient’s clinical history, the anatomical pattern of recurrence, the morphological overlap, and the concordant mesonephric-like immunophenotype favored recurrence of the ovarian MLA rather than an independent primary gynecological carcinoma arising at the vaginal cuff.

Molecular analysis was available only for the vaginal cuff recurrence and demonstrated a KRAS p.G12D alteration. Although this alteration is compatible with the molecular spectrum reported in MLA, it is not specific for this tumor type and, in the absence of molecular testing of the ovarian and bladder tumors, cannot establish clonality.

Nevertheless, considering the overall clinical course, including the primary ovarian tumor followed by a bladder lesion of gynecological origin and a subsequent vaginal cuff recurrence, together with the overlapping morphological features, concordant immunohistochemical profiles across the available specimens, and supportive molecular findings, the integrated clinicopathological assessment strongly favored a common tumor origin. Taken together, these findings support the interpretation of a primary ovarian mesonephric-like adenocarcinoma with subsequent recurrences involving the bladder and vaginal cuff.

The case was discussed at the multidisciplinary tumor board, and the therapeutic decision was to administer chemotherapy with paclitaxel (175 mg/m^2^, day 1) plus carboplatin (AUC 5, day 1) at 21-day intervals.

As of August 2026, the patient is receiving chemotherapy with paclitaxel (175 mg/m^2^, day 1) and carboplatin (AUC 5, day 1). Imaging assessment after four cycles demonstrated a favorable treatment response, with an approximately 50% reduction in the size of the vaginal tumor mass (Figure 9). Current clinical and imaging evaluations show no evidence of new lesions at other sites.

The key clinical, pathological, and therapeutic events throughout the approximately six-year disease course are summarized in Figure 10.

## 3. Discussion

Mesonephric-like adenocarcinoma is a recently recognized entity included in the fifth edition of the WHO Classification of Female Genital Tumors in 2020 [4]. Its designation reflects histopathological, immunohistochemical, and molecular similarities to mesonephric carcinoma arising at other sites, most commonly the cervix. Although conventional mesonephric carcinoma arises from mesonephric remnants, the pathogenesis of meso-nephric-like carcinoma remains unclear. Some authors propose that ovarian MLA arises from mesonephric remnants in the paraovarian region, whereas others consider it a Müllerian carcinoma that has undergone mesonephric differentiation. The latter hypothesis is supported by the frequent association of MLA with endometriosis, serous borderline tumors, and low-grade serous carcinoma [5,6,7]. Extensive molecular analyses have demonstrated shared alterations, including KRAS and NRAS mutations, among associated lesions [8,9].

The age of diagnosis varies considerably. Most patients are postmenopausal and are diagnosed between 52 and 76 years of age [10,11], although younger patients have also been reported [12]. Clinical presentation usually includes abdominal or pelvic pain or abnormal uterine bleeding; some patients may be asymptomatic when the disease is detected at an early stage [13]. Our patient presented with a ruptured, cystic ovarian tumor and abdominal pain, a clinical presentation also reported in other patients, particularly those with large cystic tumors [11,14]. Peritoneal washings are routinely performed in ovarian tumors to assess for malignant cells, although interpretation may be challenging in Müllerian and mesonephric lesions [15].

Specific radiological features have not been well characterized in the literature. Some case reports describe low signal intensity on T2-weighted MRI, high diffusion-weighted imaging signal intensity, and a smooth solid component without irregular papillary projections [16,17]. In contrast, other studies have found that a multilocular architecture with intermixed solid components is nonspecific on MRI [18].

Macroscopically, ovarian MLA lacks distinctive features. Tumors are usually unilateral and vary in size, ranging from 4 to 32 cm [12]. The cut surface is frequently cystic, with solid yellowish areas. Similar to our case, papillary projections may be absent [5].

Microscopically, ovarian MLA resembles mesonephric carcinoma of the cervix and may exhibit diverse architectural patterns, including tubular, pseudoendometrioid, glomeruloid, papillary, ductal, retiform, solid, corded, and sieve-like patterns [19]. Eosinophilic secretions are often present within glandular lumina. Consequently, the differential diagnosis can be challenging because MLA may mimic endometrioid carcinoma, low-grade serous carcinoma, or clear cell carcinoma [3]. In our case, the primary ovarian tumor was initially diagnosed as low-grade serous carcinoma. The first bladder recurrence was interpreted as urothelial carcinoma with squamous and glandular differentiation, whereas the subsequent partial cystectomy specimen was considered a carcinoma of gynecological origin, most likely endometrioid carcinoma. Cytological features may aid diagnosis: tumor cells are often cuboidal or columnar and relatively monomorphic, with moderate nuclear pleomorphism, nuclear grooves, and conspicuous mitotic activity. As in our case, hobnailing may also be present, potentially leading to confusion with clear cell carcinoma. Notably, metaplastic changes, such as ciliated, mucinous, or squamous differentiation, do not support a diagnosis of MLA [16].

Immunohistochemistry is crucial for diagnosing this rare ovarian tumor subtype. Mills et al. [3] conducted an extensive review of diagnostic markers and proposed a recommended immunohistochemical panel. They considered TTF1, GATA3, ER, and PR essential markers. A comprehensive literature review showed that these tumors are typically positive for TTF1 and GATA3 and negative for hormone receptors (ER and PR) [5,20]. PAX8 is frequently expressed in MLA and can support a gynecologic origin in the appropriate morphological and immunohistochemical context [21]. CD10 is frequently used as an additional marker because it highlights the lumina of the tumor glands. Other markers may be useful for differential diagnosis. Endometrioid carcinoma is probably the most frequent misdiagnosis; testing for ER and PR can help distinguish the entities, as negative or very weak staining is uncommon in low-grade endometrioid carcinomas [22]. Low-grade and high-grade serous carcinomas should also be considered in differential diagnosis. p53 is useful because aberrant expression is characteristic of high-grade serous carcinoma, whereas MLA usually shows a wild-type pattern. Strong nuclear WT1 expression is also uncommon in MLA and can support a diagnosis of low- or high-grade serous carcinoma. However, caution is warranted because focal WT1 positivity has been reported, potentially reflecting tumor heterogeneity [23]. MLA may show nuclear hobnailing and clear cell features resembling clear cell carcinoma. Our case showed similar features, prompting evaluation with markers associated with clear cell carcinoma. Napsin A was strongly positive in the clear cell-like areas of the tumor. Several studies have reported Napsin A expression in MLA, with positivity reported in approximately 40.6% and 22% of cases, respectively [24,25]. Napsin A expression has been reported in a substantial subset of MLAs and should not be used in isolation to diagnose clear cell carcinoma. Occasionally, MLA may exhibit spindle-cell architecture, raising the possibility of carcinosarcoma. Several case reports have described MLA with sarcomatous differentiation, including chondroid or rhabdomyosarcomatous components, particularly in recurrent disease [26,27]. One unusual case described synchronous bilateral ovarian MLA arising in association with high-grade adenosarcoma and endometriosis [28].

Our case showed an association between MLA and a serous borderline tumor. Numerous case reports have described associations with endometriosis, cystadenomas, adenofibromas, serous borderline tumors, low-grade serous carcinoma, or mucinous tumors [2]. Dundr et al. [29] demonstrated that the MLA and serous borderline tumor components shared similar molecular alterations and hypothesized that the borderline component could represent a precursor lesion, supporting a Müllerian origin for both components. However, other authors have proposed that distinct molecular alterations may account for the different phenotypes within mixed tumors [9]. This hypothesis has been supported by various studies [29,30,31]. Association with mucinous borderline tumors or mucinous carcinoma is extremely rare, with only a few reported cases. Smith et al. [32] explored potential mechanisms of transdifferentiation or tumorigenesis in these settings. They studied nine cases and found that, although one case showed clonal similarities between the two components, other cases demonstrated lineage-specific differentiation. Another study described an association of three tumor components—MLA, extensive endometriosis, and endometrioid carcinoma—with shared KRAS G12V and ARID1A mutations and gains in the 1q21.3 region [33]. Association with adenofibroma has also been documented in an unusual case of synchronous ovarian and endometrial MLA potentially arising from an endometrioid adenofibroma and sharing a KRAS G12D mutation [34]. Another report described MLA associated with bilateral endometriotic cysts that shared a KRAS p.G12V mutation [35]. Endometriosis is frequently associated with ovarian MLA [36,37,38,39]. Low-grade serous carcinoma may occur concurrently with MLA but MLA is also frequently misdiagnosed as low-grade serous carcinoma, potentially delaying appropriate oncological treatment [40].

Table 2 summarizes recently documented molecular alterations in ovarian and extra-ovarian MLA. Molecular analysis in our patient was performed using next-generation sequencing (NGS) on tissue obtained from the vaginal cuff recurrence. The analysis identified a KRAS G12D variant. Molecular testing was not performed on the primary ovarian tumor or the bladder recurrence; therefore, molecular clonality among the three lesions could not be definitively established. Nevertheless, the clinical course, morphological features, and consistent immunohistochemical profiles of all three tumors were consistent with recurrent ovarian MLA. Several studies have demonstrated that KRAS mutations are involved in the development of ovarian and endometrial MLA [29,41,42]. Koh et al. [43] reported that approximately 82.1% of reviewed cases harbored pathogenic KRAS mutations, with p.G12D accounting for approximately 43% of these mutations. Interestingly, in recurrent and metastatic disease, KRAS alterations may be similar to those identified in the primary tumor [30,41]. Some authors have investigated the potential prognostic significance of different KRAS mutation sites in gynecological mesonephric-like tumors. In ovarian tumors, 5 of 11 patients experienced recurrence regardless of the KRAS mutation site [44].

Molecular testing also identified a PTEN variant resulting in a premature stop codon and a predicted truncated protein, suggesting probable loss of PTEN function. The occurrence of PTEN, PIK3CA, and CTNNB1 alterations in subsets of MLA has been interpreted as additional evidence supporting a possible Müllerian origin [30,45]. Some authors have proposed that MLA may represent a variant of endometrioid carcinoma, particularly in tumors harboring PIK3CA mutations [45]. Interestingly, PTEN or PIK3CA alterations may provide additional support for a diagnosis of MLA when distinguishing it from mesonephric carcinoma arising at a more typical site, such as the cervix [20].

There are currently no standard treatment guidelines for this rare histological subtype. In our patient, treatment decisions at the different stages of the disease reflected the pathological diagnosis and clinical information available at each time point. Following the initial diagnosis of FIGO stage IC2 low-grade serous ovarian carcinoma, the patient received six cycles of paclitaxel plus carboplatin. When the bladder lesion subsequently developed, its initial pathological interpretation raised the possibility of a primary urothelial carcinoma; however, the clinical and oncology teams considered recurrence of the ovarian carcinoma, and the patient received six cycles of gemcitabine plus carboplatin. Following the vaginal cuff recurrence and integrated reassessment of the ovarian, bladder, and vaginal tumors, the diagnosis was revised to ovarian MLA with subsequent recurrences. The case was discussed at the multidisciplinary tumor board, and treatment with paclitaxel plus carboplatin was initiated, resulting in an approximately 50% reduction in the vaginal tumor mass after four cycles. Radical surgery is commonly performed, particularly for localized disease [48]. Adjuvant chemotherapy remains one of the principal treatment options. However, there is insufficient data to support a specific ovarian cancer treatment regimen, such as carboplatin plus paclitaxel. Nevertheless, case reports and small case series suggest that MLA may respond to platinum-based doublet chemotherapy, including in the recurrent setting [11,46,49]. Other systemic treatments have limited supporting evidence. Endocrine therapy and PARP inhibitors may have limited utility because MLAs are generally hormone receptor-negative and often lack BRCA1/BRCA2 mutations or homologous recombination deficiency [41,49,50]. Immune-checkpoint inhibition could theoretically be considered in tumors with established predictive biomarkers such as mismatch repair deficiency or microsatellite instability; however, these alterations appear to be uncommon in MLA, and MLA-specific clinical evidence remains limited [50]. Radiotherapy has not been well studied in ovarian MLA, although it has been used in uterine MLA with some reported therapeutic benefit [51]. Regarding predictive biomarkers, one study evaluated HER2 and FOLR1 expressions in 21 MLA cases from different gynecological sites, including five primary ovarian MLAs. Because of the small cohort and predominantly negative results for both biomarkers, further studies are required to determine their clinical utility [52].

As described above, somatic KRAS mutations may represent potential therapeutic targets through inhibition of the MAPK signaling pathway. MAPK-pathway inhibition is being investigated in gynecologic tumors. An earlier study of avutometinib plus defactinib in recurrent low-grade serous ovarian carcinoma reported an overall response rate of 46% (NCT04625270, https://clinicaltrials.gov/study/NCT04625270 (accessed on 15 August 2026)), whereas the ongoing phase II trial NCT05787561 (https://clinicaltrials.gov/study/NCT05787561, accessed on 15 August 2026) specifically evaluates this combination in advanced or recurrent mesonephric gynecologic cancer and, as of August 2026, has no posted efficacy results. For uncommon subtypes with poor prognosis, such as MLA, there is an urgent need for effective targeted therapies. Other investigators have characterized the molecular landscape of ovarian MLA and proposed dual inhibition of the MAPK and PI3K pathways to enhance therapeutic response and personalize treatment [53]. Considering the frequency of PIK3CA mutations and KRAS mutations, Kasuya et al. [54] evaluated trametinib, a MEK1/MEK2 inhibitor, and omipalisib, a PI3K/mTOR pathway inhibitor, in MLA. In vivo studies using patient-derived xenograft mouse models showed that the combination produced an antitumor effect similar to that of carboplatin treatment.

Although earlier recognition of MLA would not necessarily have resulted in a different systemic treatment regimen, given the absence of established MLA-specific treatment guidelines, it may have influenced clinical surveillance and the interpretation of subsequent lesions. Awareness of the reported aggressive behavior and recurrence pattern of MLA could support individualized follow-up and a lower threshold for investigating new lesions as possible metastatic or recurrent disease. In the present case, knowledge of the original tumor as an MLA might also have raised earlier consideration of secondary bladder involvement rather than a new primary urothelial malignancy.

Surveillance of these rare ovarian tumors is difficult to standardize. Most oncologists follow guidelines based on the primary tumor site and histological diagnosis. Because these tumors are aggressive and have a high risk of recurrence, improved surveillance strategies may be warranted. Available data indicate a prolonged interval to first recurrence [46], similar to the approximately five-year interval observed in our patient. However, once recurrence occurs, progression may be rapid, underscoring the importance of appropriate surveillance after initial treatment. Among reported recurrent ovarian MLAs, the lung appears to be a common site of distant recurrence [46,55]. A recently reported case described lung metastasis presenting as carcinosarcoma arising from ovarian MLA, emphasizing the importance of considering a history of ovarian cancer when evaluating lung tumors [56]. The reported propensity for pulmonary metastasis highlights the importance of awareness of the lung as a potential site of recurrence during clinical and radiological follow-up. Other reported metastatic sites include the spleen, peritoneum, lymph nodes, colorectum, bladder, and brain [4,39].

The significance of the present case extends beyond the rarity of ovarian MLA and lies particularly in the sequence of diagnostic challenges encountered across three anatomically distinct sites. The primary ovarian tumor was initially interpreted as low-grade serous carcinoma, whereas the subsequent bladder lesion raised the possibility of a primary urothelial carcinoma and was later considered a gynecological carcinoma, and the vaginal cuff lesion ultimately prompted recognition of the mesonephric-like phenotype and retrospective reassessment of the disease course. Bladder involvement is of particular diagnostic interest because the extrinsic pattern of invasion and preservation of the overlying urothelium, together with the morphological and immunohistochemical findings, favored secondary involvement rather than a primary bladder carcinoma. This case also illustrates the potential limitations of relying on individual immunohistochemical markers: GATA3 expression may suggest urothelial differentiation, while Napsin A positivity in areas with clear-cell/hobnail morphology may raise the possibility of clear cell carcinoma. Conversely, the combined assessment of TTF1 and GATA3 expression, luminal CD10 staining, ER negativity, and the overall morphological pattern can support mesonephric-like differentiation in the appropriate clinicopathological context. The identification of a KRAS p.G12D alteration in the vaginal cuff lesion provides additional support for a molecular profile compatible with MLA, although this alteration is not specific for MLA and, in the absence of molecular testing of the other lesions, cannot establish clonality. Taken together, this case emphasizes the importance of integrating clinical history, anatomical pattern of tumor involvement, morphology, immunohistochemistry, and molecular findings to avoid misclassification of MLA, particularly when recurrent disease involves the urinary tract or exhibits morphological and immunophenotypic overlap with other gynecological or urothelial malignancies.

Previous studies have demonstrated that ovarian MLA has a substantial propensity for recurrence and distant metastasis, with the peritoneum, lungs, and liver among the reported sites of recurrent disease (Table 3).

The distinctive feature of the present case is therefore not recurrence itself, but rather the sequential involvement of anatomically and diagnostically challenging sites. In particular, extrinsic involvement of the urinary bladder mimicked a primary urothelial malignancy, while the subsequent vaginal cuff lesion provided morphological and immunohistochemical findings that prompted recognition of the mesonephric-like phenotype and reassessment of the previous tumors. This sequence highlights how the heterogeneous morphology and overlapping immunophenotype of MLA may result in different diagnoses depending on the anatomical site sampled. Accordingly, awareness of the patient’s previous ovarian tumor and integration of morphology with a panel of mesonephric and Müllerian markers are essential when evaluating subsequent lesions at unusual sites.

## 4. Conclusions

Ovarian MLA is a rare subtype of ovarian carcinoma that frequently harbors KRAS mutations and may exhibit aggressive clinical behavior. This case highlights the diagnostic pitfalls associated with this uncommon entity and the potential value of comprehensive immunohistochemical and molecular testing in establishing the diagnosis. Recurrent ovarian MLA poses a diagnostic and therapeutic challenge, particularly because comprehensive molecular testing of every recurrent tumor may not always be financially feasible or readily accessible. Consequently, molecular characterization could not be performed on all tumor recurrences in our case, which represents a limitation of the present study. Therefore, molecular clonality between the primary ovarian tumor and the subsequent bladder and vaginal lesions could not be established. Molecular profiling may also assist multidisciplinary teams in selecting treatment strategies and developing individualized surveillance plans. Further research is needed to identify effective targeted therapies for patients with recurrent or advanced ovarian MLA.

## Figures and Tables

**Figure 1:**
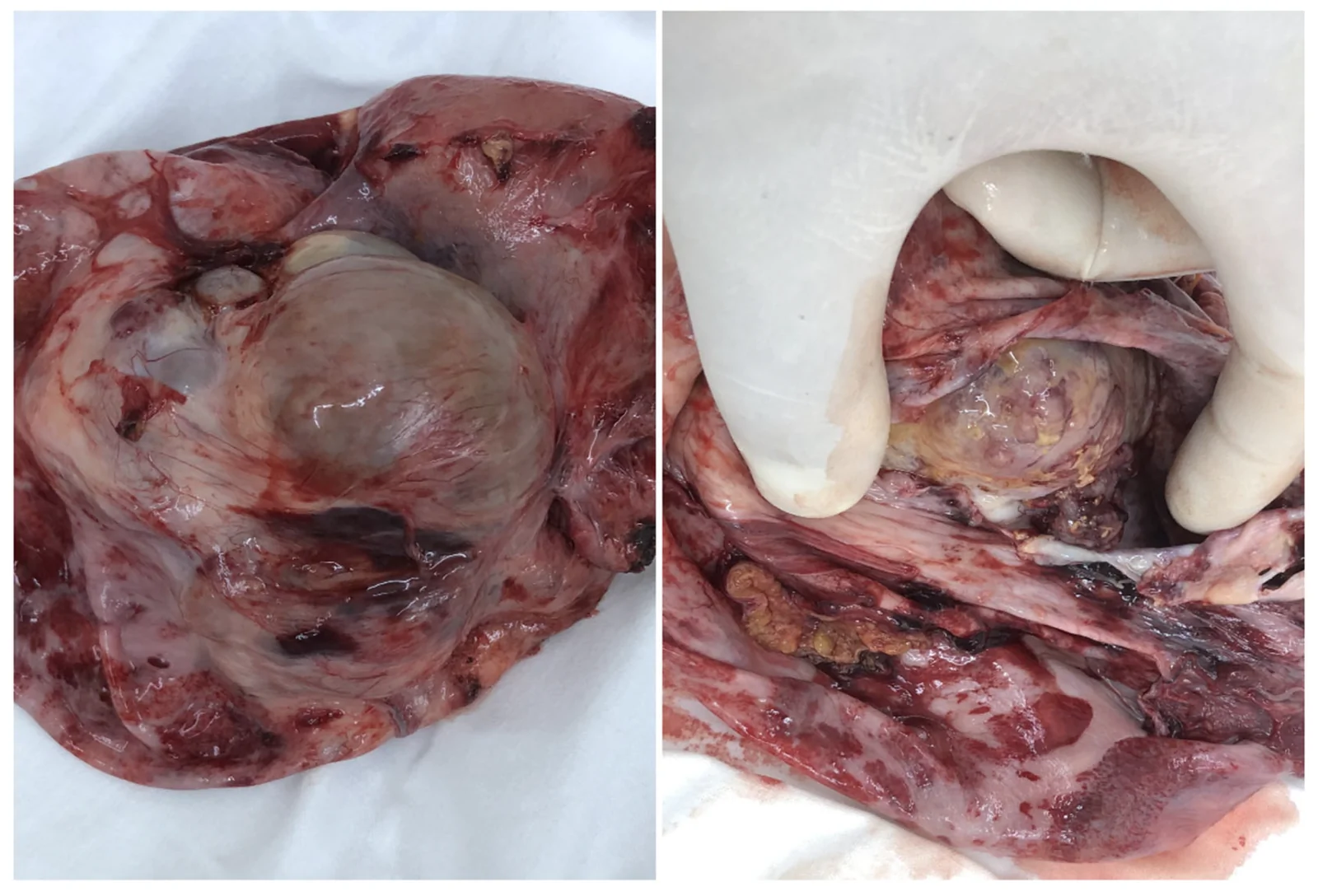
Macroscopic features of the ruptured, cystic left ovarian tumor.

**Figure 2:**
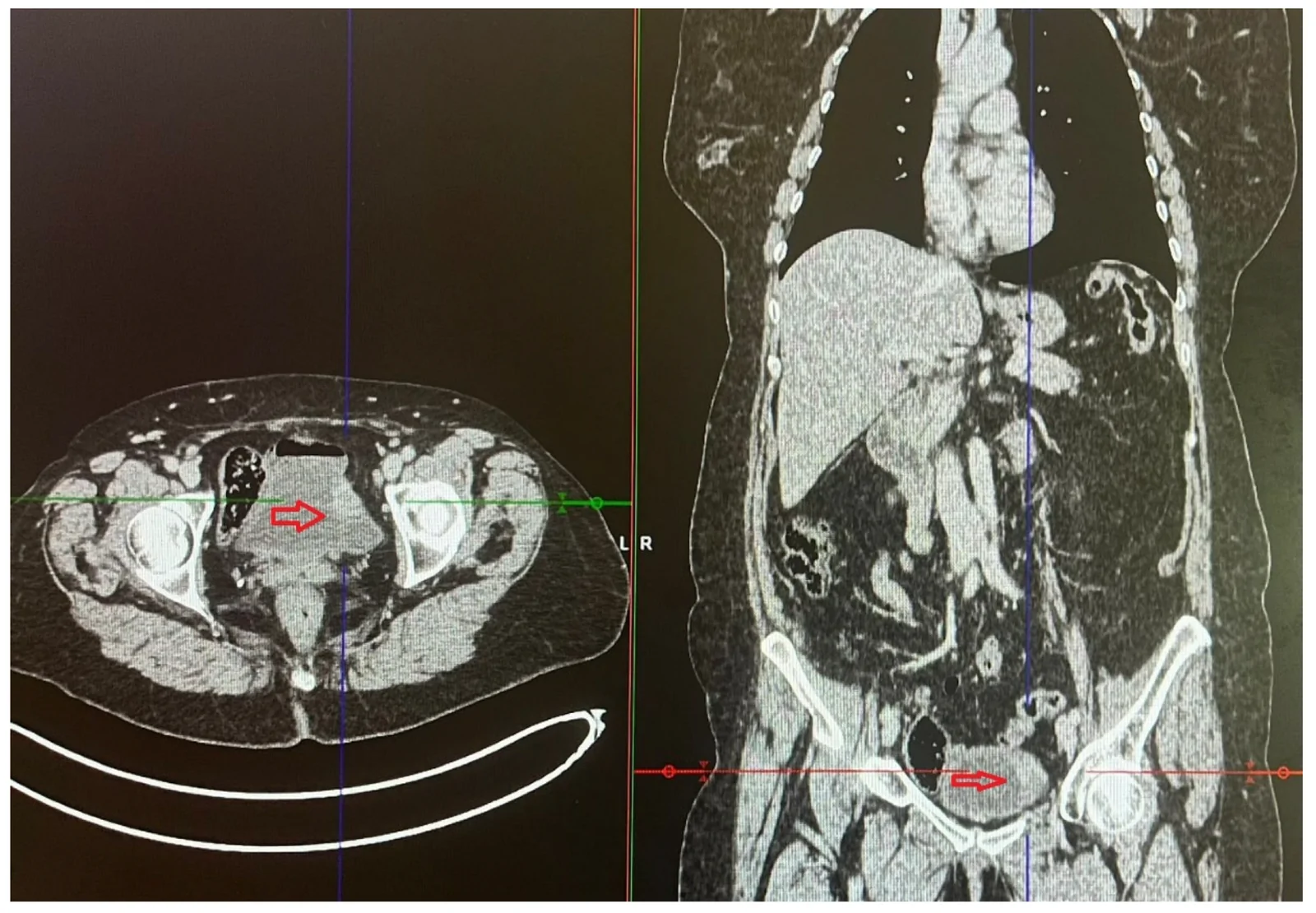
CT examination showing tumor recurrence in the bladder wall (red arrows).

**Figure 3:**
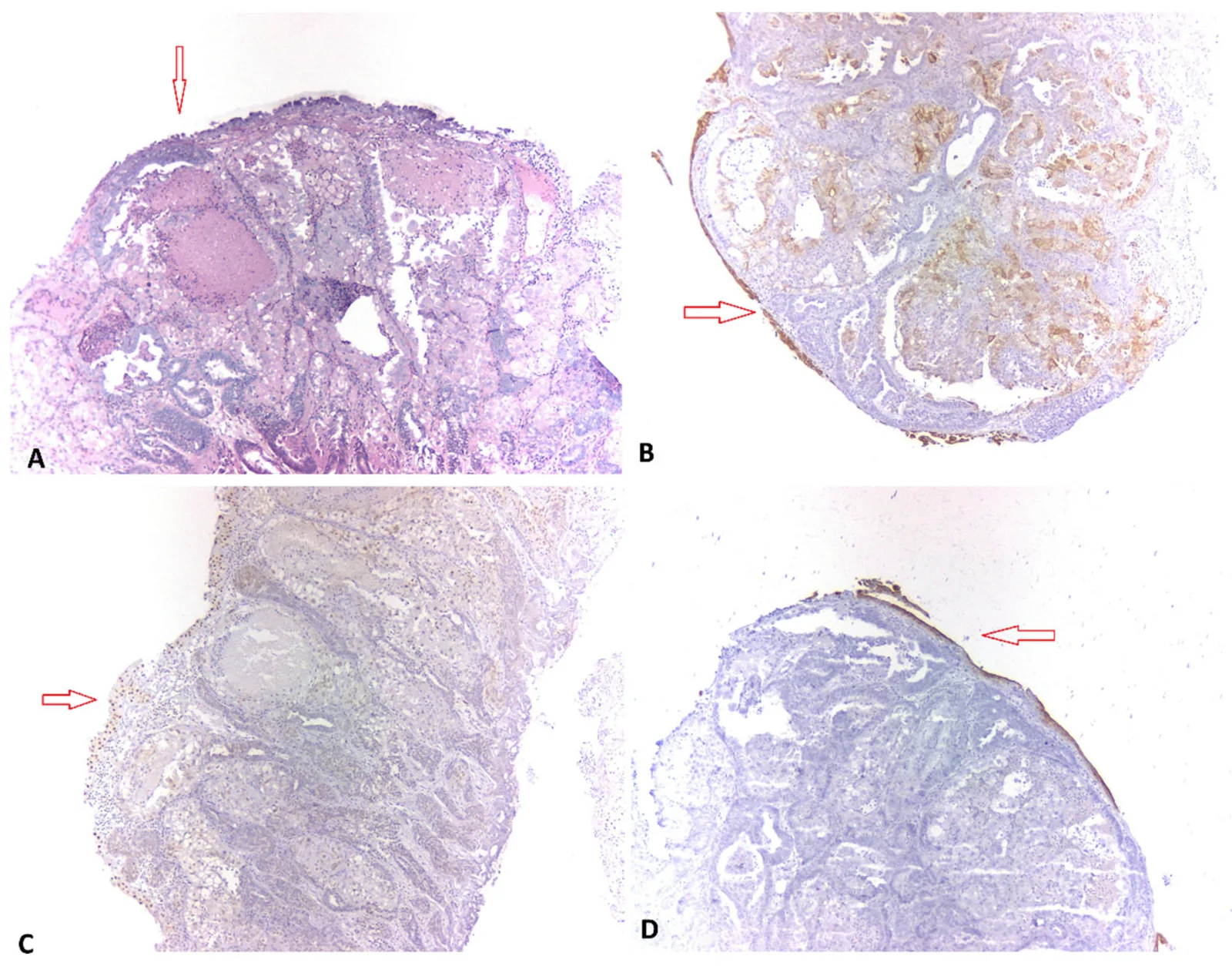
(**A**) Hematoxylin and eosin (H&E), 40× magnification: MLA with pseudoglandular architecture and clear cell features; extensive tumor necrosis is present, while the urothelium is unaffected (red arrow). (**B**) CK7, 100× magnification: membranous and cytoplasmic expression in both tumor cells and normal urothelium (red arrow). (**C**) GATA3, 100× magnification: nuclear expression in both tumor cells and urothelium (red arrow). (**D**) Uroplakin III, 100× magnification: absent expression in tumor cells, with retained positivity in normal urothelium (red arrow).

**Figure 4:**
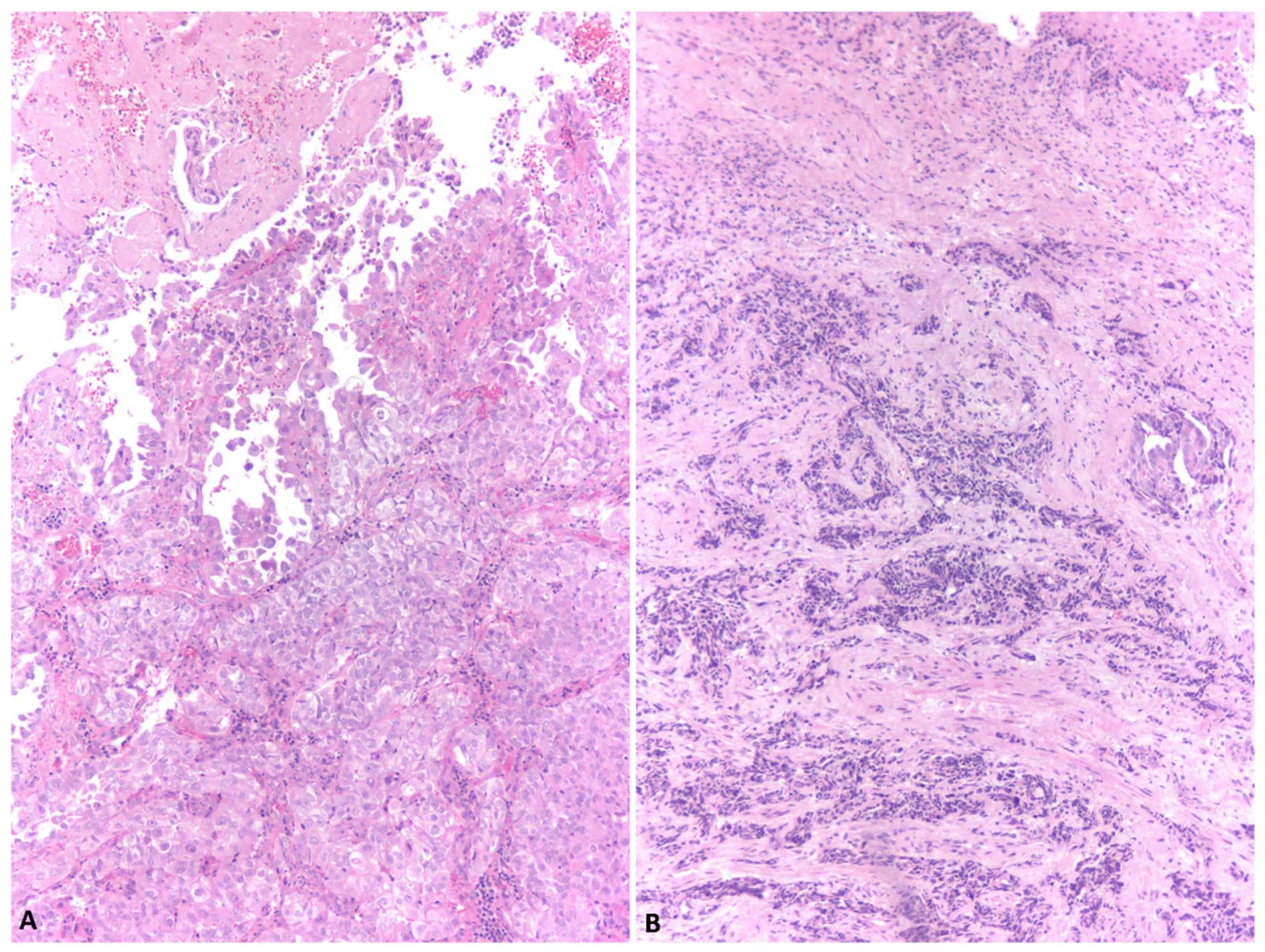
(**A**) H&E, 100× magnification: MLA with solid, clear cell, and hobnail growth patterns. (**B**) H&E, 100× magnification: MLA with pseudoglandular and solid architectural patterns.

**Figure 5:**
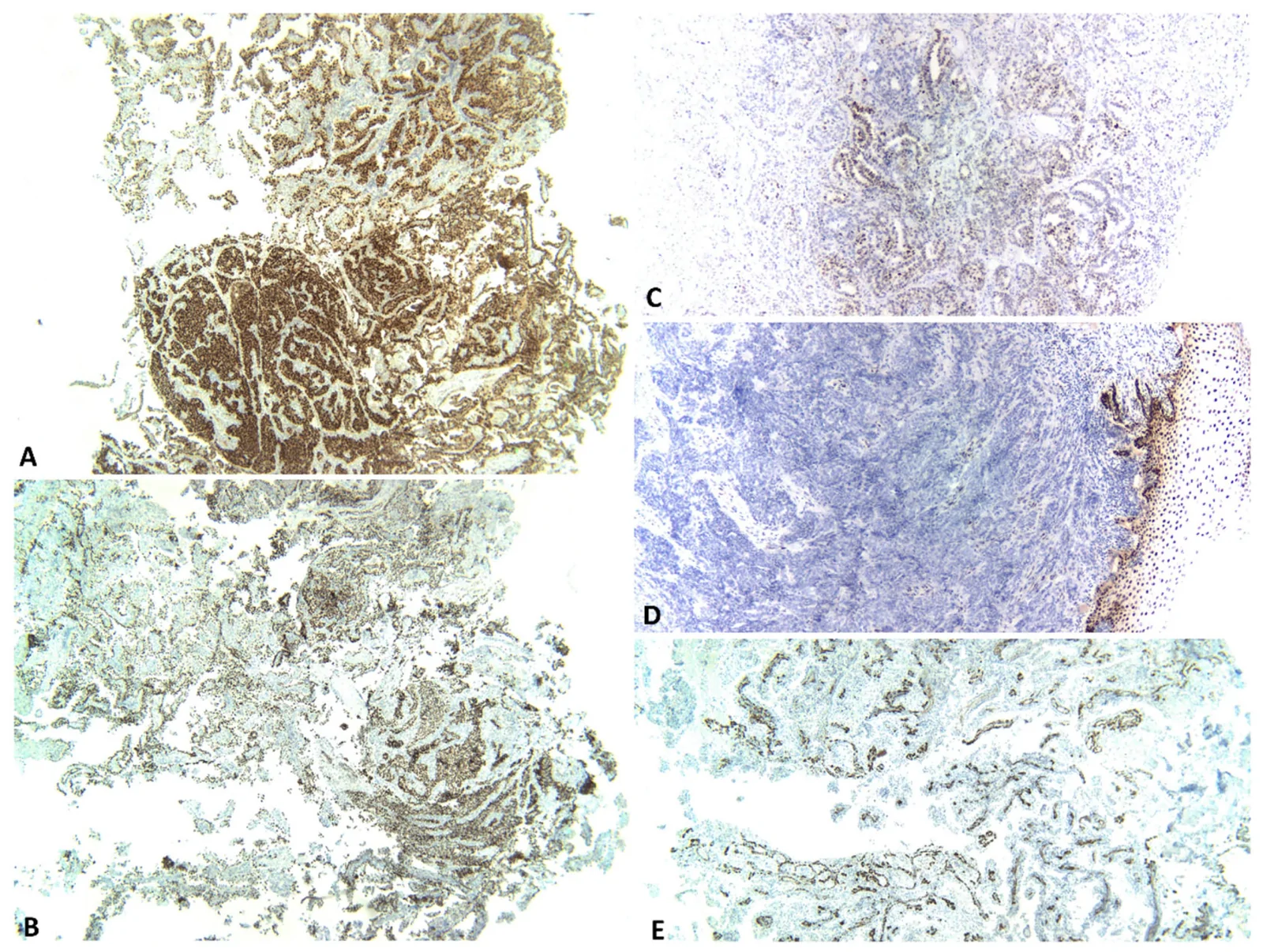
(**A**) PAX8, 40× magnification: diffuse nuclear expression in tumor cells. (**B**) TTF1, 40× magnification: diffuse nuclear expression in tumor cells. (**C**) GATA3, 100× magnification: nuclear expression in tumor cells. (**D**) ER, 40× magnification: absent nuclear expression in tumor cells, with nuclear positivity in the squamous vaginal epithelium. (**E**) WT1, 40× magnification: absent nuclear expression in tumor cells, with cytoplasmic expression in endothelial cells.

**Figure 6:**
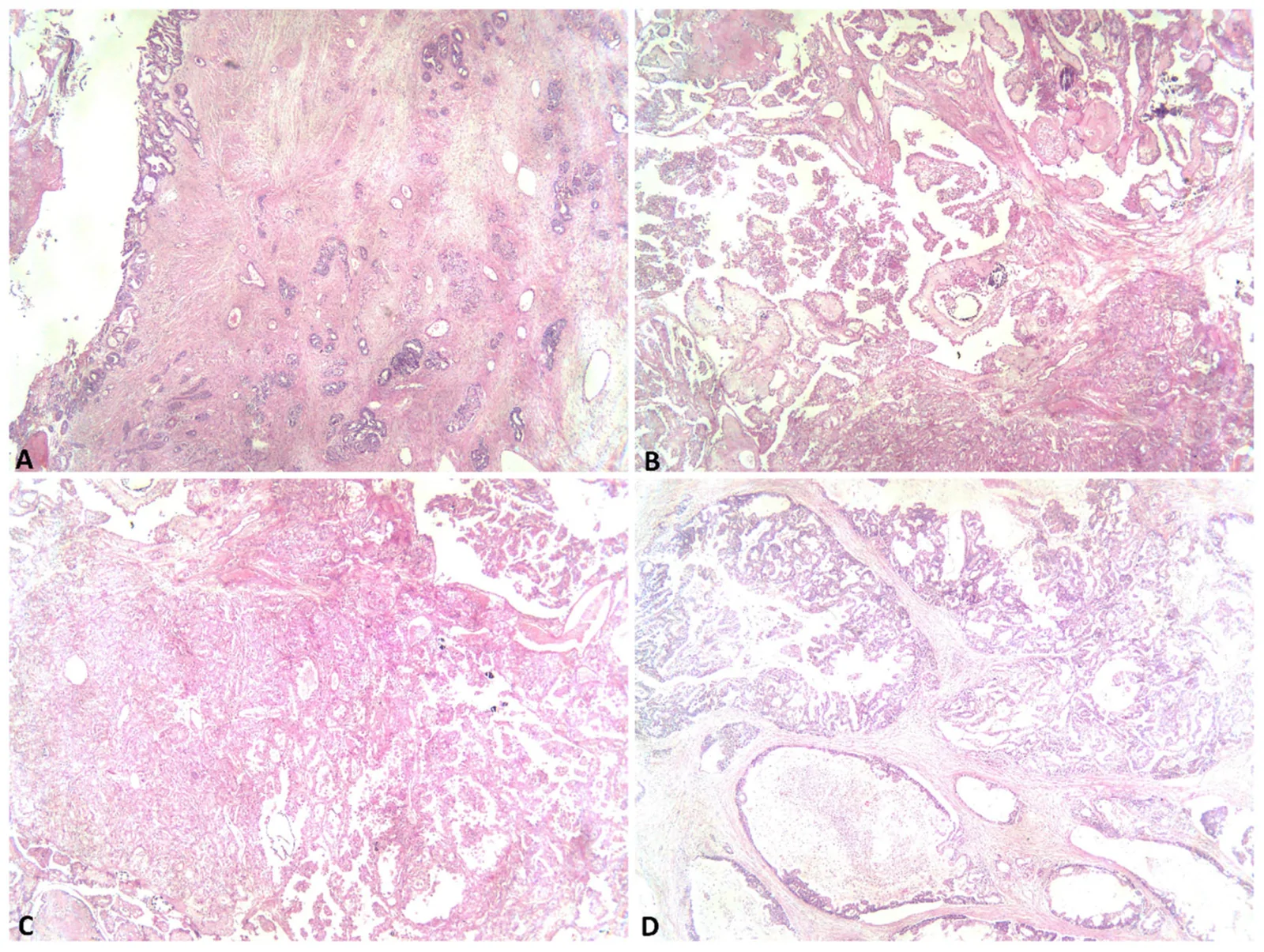
(**A**) H&E, 40× magnification: MLA with pseudoglandular and ductal architecture; an associated serous borderline tumor component is present. (**B**) H&E, 40× magnification: MLA with papillary architecture. (**C**) H&E, 40× magnification: MLA with clear cell and focal papillary architecture. (**D**) H&E, 40× magnification: MLA with glandular and ductal architecture.

**Figure 7:**
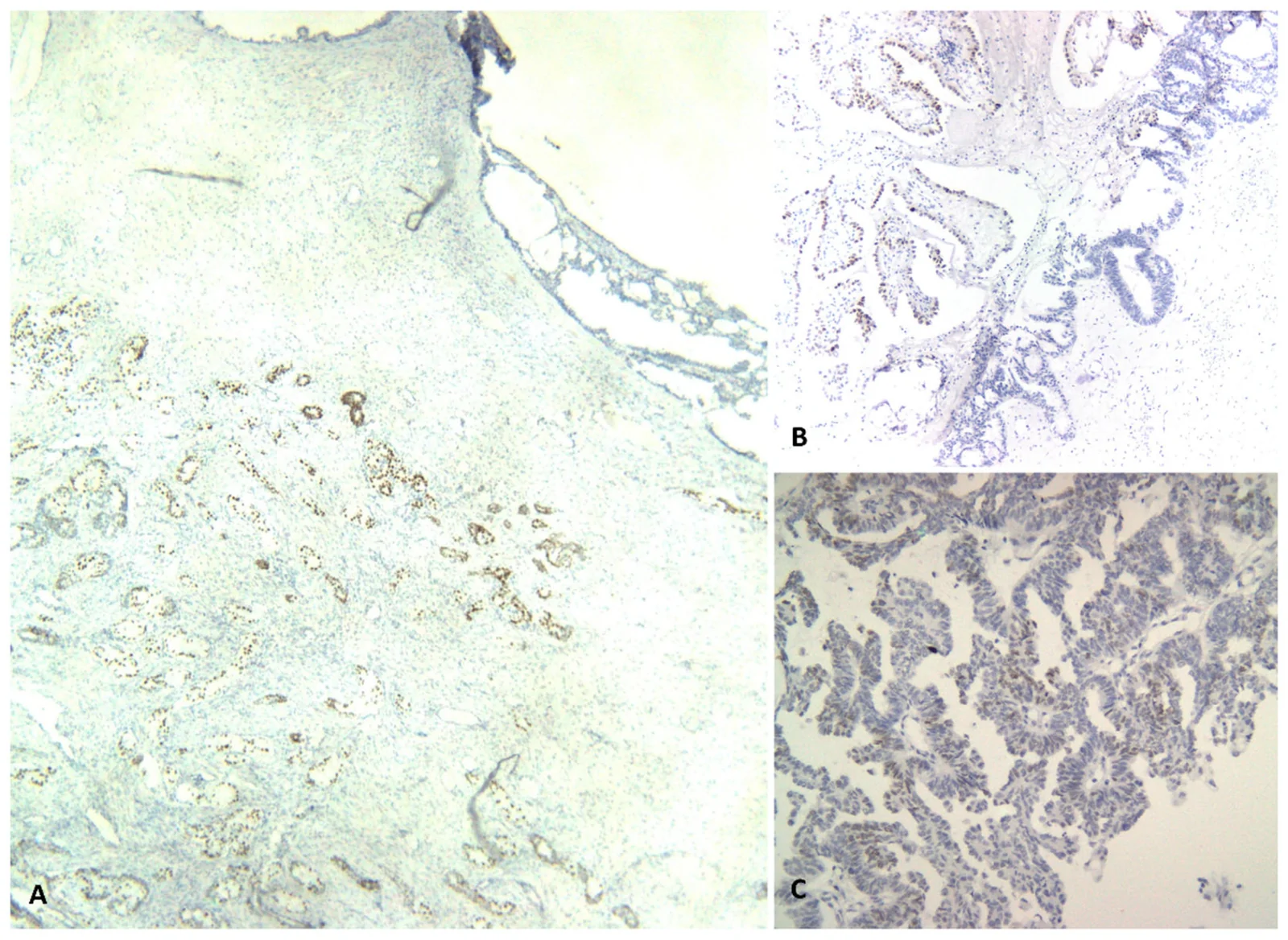
(**A**) TTF1, 40× magnification: diffuse nuclear expression in tumor cells; nuclear negativity in the serous borderline tumor component. (**B**) TTF1, 100× magnification: diffuse nuclear expression in tumor cells; nuclear negativity in the serous borderline tumor component. (**C**) GATA3, 200× magnification: focal nuclear expression in tumor cells.

**Figure 8:**
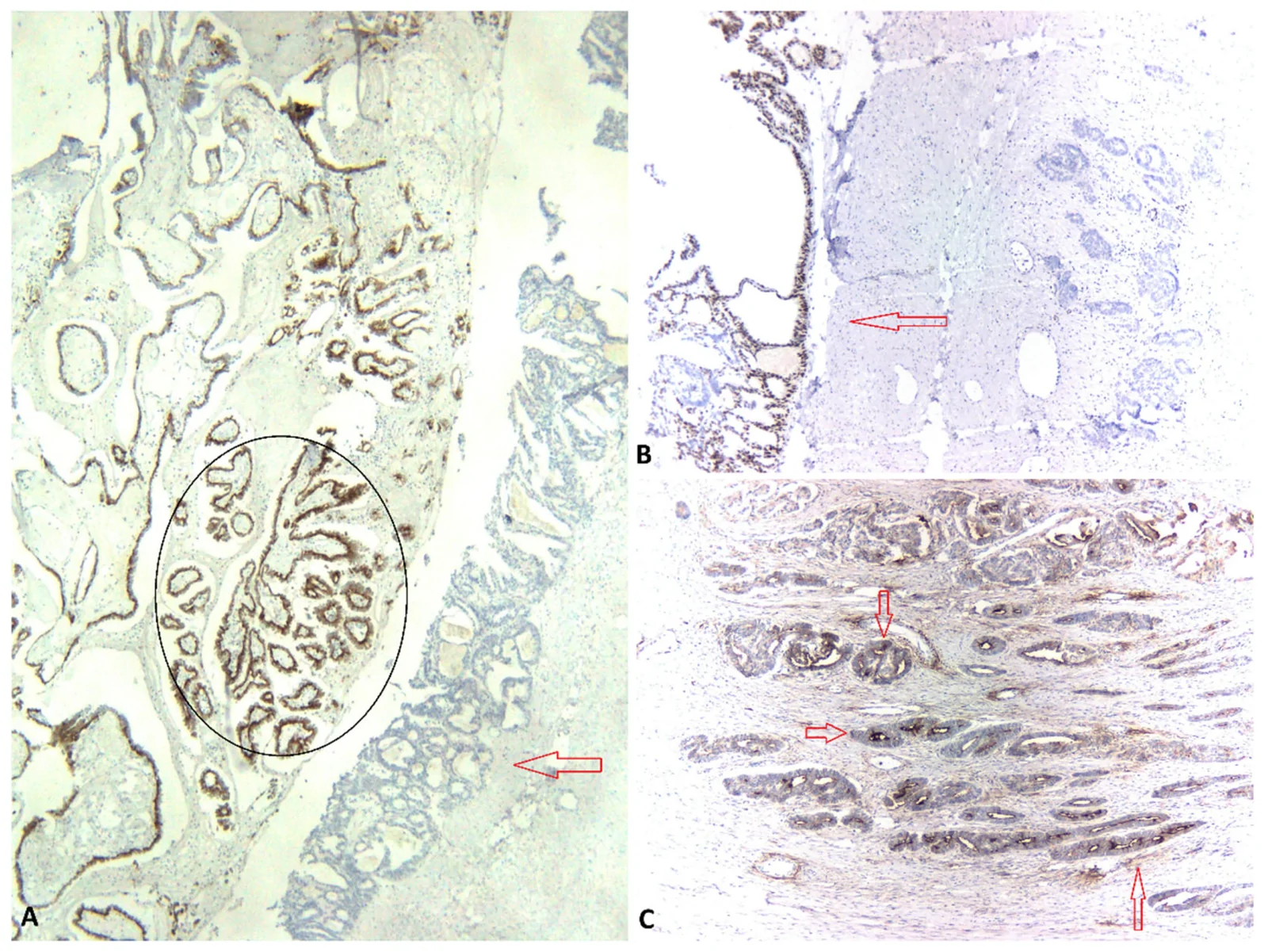
(**A**) Napsin A, 40× magnification: diffuse cytoplasmic expression in tumor cells with clear cell and hobnail features (black circle); cytoplasmic negativity in the serous borderline tumor component (red arrow). (**B**) ER, 40× magnification: absent expression in tumor cells; nuclear positivity in the serous borderline tumor component (red arrow). (**C**) CD10, 100× magnification: diffuse luminal membranous expression in MLA glands and ducts (red arrows).

**Figure 9:**
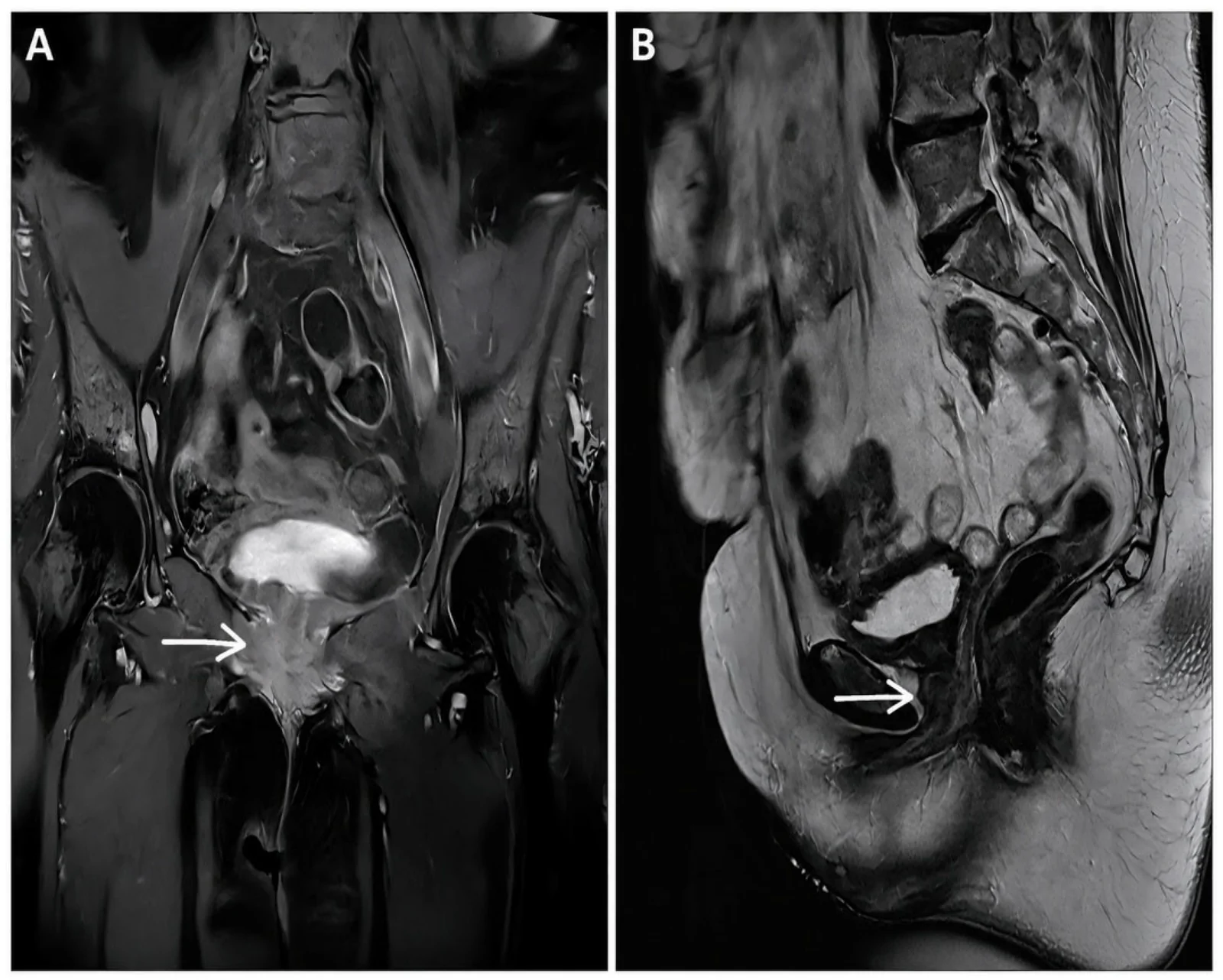
(**A**,**B**) Magnetic resonance imaging (MRI) demonstrates an approximately 50% reduction in the size of the vaginal tumor following four cycles of chemotherapy (white arrows).

**Figure 10:**
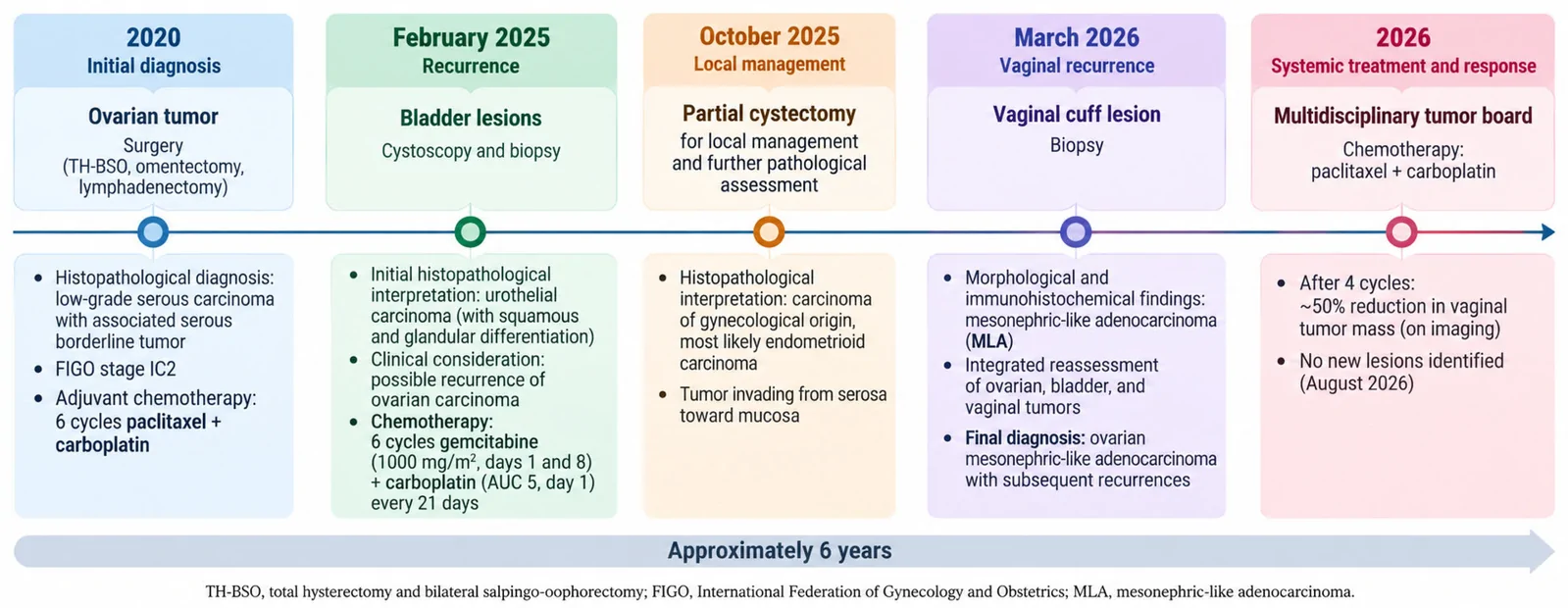
Timeline of the clinical, pathological, and therapeutic course. The timeline summarizes the evolution from the initial ovarian tumor diagnosed in 2020, through the bladder lesions and partial cystectomy in 2025, to the vaginal cuff recurrence, integrated pathological reassessment, final diagnosis of ovarian mesonephric-like adenocarcinoma (MLA), and subsequent systemic treatment and response in 2026.

**Table 1:** Morphological and immunohistochemical comparison of the primary ovarian tumor and recurrences with the main differential diagnoses of ovarian mesonephric-like adenocarcinoma, including urothelial carcinoma.

Feature	Primary Ovarian Tumor	Bladder Recurrence	Vaginal Cuff Recurrence	Low-Grade Serous Carcinoma (LGSC)	Endometrioid Carcinoma	Clear Cell Carcinoma (CCC)	High-Grade Serous Carcinoma (HGSC)	Urothelial Carcinoma
Morphology	Predominantly pseudoglandular/ductal architecture; focal papillary, solid, and clear-cell areas; associated serous borderline tumor	Pseudoglandular architecture, clear-cell features, and extensive necrosis; overlying urothelium unaffected	Poorly differentiated carcinoma with solid, pseudoglandular, clear-cell, and hobnail patterns	Papillary/micropapillary architecture; relatively uniform nuclei; psammoma bodies may be present	Glandular architecture resembling endometrial endometrioid carcinoma; squamous differentiation may occur	Tubulocystic, papillary, and solid architecture; clear and hobnail cells	Papillary, solid, and slit-like architecture; marked nuclear atypia and high mitotic activity	Papillary, nested, solid, or infiltrative growth; urothelial differentiation with variable glandular or squamous differentiation may occur.
PAX8	Not tested	Positive	Positive	Positive	Positive	Positive	Positive	Usually negative
TTF1	Positive	Not tested	Positive	Usually negative	Usually negative	Usually negative	Usually negative	Negative/non-characteristic
GATA3	Positive	Positive on initial biopsy; negative on partial cystectomy	Positive	Usually negative	Usually negative	Usually negative	Usually negative	Frequently positive
CD10	Positive, luminal membranous	Not tested	Not tested	Usually negative/non-characteristic	Variable	Usually negative/non-characteristic	Usually negative	Non-characteristic
Napsin A	Positive, particularly in clear-cell/hobnail areas	Not tested	Not tested	Usually negative	Usually negative	Frequently positive	Usually negative	Usually negative
ER	Negative	Negative	Negative	Frequently positive	Frequently positive	Usually negative	Frequently positive	Usually negative
WT1	Not tested	Negative	Negative	Usually positive	Usually negative	Usually negative	Usually positive	Negative
p53	Wild-type pattern	Wild-type pattern	Wild-type pattern	Usually wild-type	Usually wild-type	Usually wild-type	Usually aberrant/mutation-type	Variable; aberrant pattern may occur
CK7	Not tested	Positive	Positive	Positive	Positive	Positive	Positive	Frequently positive
CK20	Not tested	Negative	Negative	Usually negative	Usually negative	Usually negative	Usually negative	Variable; often positive
Uroplakin III	Not tested	Negative	Negative	Negative	Negative	Negative	Negative	Positive in a subset; supports urothelial differentiation when present
Key findings favoring MLA	TTF1+/GATA3+, luminal CD10+, ER− phenotype combined with characteristic pseudoglandular/ductal morphology	PAX8 positivity, ER/WT1 negativity, absence of urothelial involvement, and clinicopathological concordance with primary tumor	PAX8+/TTF1+/GATA3+, ER−/WT1− phenotype with concordant morphology	Mesonephric marker profile and ER negativity favor MLA; p53 wild-type alone is not discriminatory	Mesonephric marker profile and ER negativity favor MLA	Important mimic because of Napsin A positivity and clear/hobnail morphology; overall morphology and mesonephric marker profile favor MLA	WT1 negativity and wild-type p53 pattern argue against conventional HGSC	PAX8 positivity with uroplakin III and CK20 negativity supports gynecological origin; GATA3 positivity alone is not specific for urothelial carcinoma.
Integrated interpretation	Primary ovarian MLA	Consistent with recurrence of ovarian MLA	Consistent with recurrence of ovarian MLA	Less supportive of LGSC	Less supportive of endometrioid carcinoma	CCC requires exclusion by integrated morphology and IHC	Less supportive of HGSC	Less supportive of urothelial carcinoma

Note: The integrated interpretation of the bladder and vaginal lesions as recurrences is based on the combined clinical, morphological, and immunohistochemical findings.

**Table 2:** Selected representative studies of molecular alterations in ovarian and extrauterine MLA.

Study and Cohort	KRAS/MAPK Alterations	Other Recurrent Alterations	Copy-Number Findings
Mirkovic et al., 2018 [45] 7 MLA cases (4 ovarian; 3 uterine corpus)	KRAS mutations in 7/7 (G12D, *n* = 4; G12V, *n* = 3)	PIK3CA activating mutations in 3/7; no PTEN, ARID1A, or TP53 alterations	1q gain in 5/7; chromosome 10 gain in 4/7; chromosome 12 gain in 3/7
Lin et al., 2020 [41] 20 MLA cases (4 ovarian)	KRAS mutations in 18/20 overall (G12V, G12D, G12A, G12C)	ARID1A 5/20; PIK3CA 4/20; CTNNB1 3/20; TP53, MLL2, CDKN2A, BCOR each 2/20	1q gain in 19/20; chromosome 10 gain in 12/20; chromosome 20 gain in 12/20
da Silva et al., 2021 [30] 28 MLA cases (15 ovarian; 13 endometrial)	KRAS mutations in 25/28 overall; 2 KRAS-wild-type ovarian tumors had NRAS hotspot mutations	PIK3CA 8/28; CTNNB1 4/28; PTEN 3/13; other alterations included ARID1A and pathway-related genes	1q gain in 89%; chromosome 10 gain in 43%; chromosome 12 gains were more frequent in ovarian MLA
Arslanian et al., 2022 [42] 3 MLA cases (2 ovarian; 1 uterine)	KRAS G12D or G12V in 3/3	PIK3CA H1047Q in 1/3	Not systematically reported
Praiss et al., 2024 [46] 81 gynecologic MA/MLA cases (28 ovarian MLA); 26 underwent MSK-IMPACT	MAPK-pathway alterations in 25/26 tested tumors; the study supports high prevalence of KRAS/MAPK alterations	Study-level sequencing was heterogeneous; detailed site-specific gene frequencies should not be inferred from the overall cohort	Not summarized as a site-specific ovarian CNV frequency in the primary report
Qin et al., 2025 [47] 8 MLA cases (ovary and endometrium)	Pathogenic KRAS mutations in 4/8; pathogenic NRAS p.Q61K in 1/8	No additional recurrent alteration suitable for frequency reporting from the abstract	Not reported in the study abstract
Shi et al., 2026 [14] 9 MLA cases; 7 underwent NGS	KRAS mutations in 5/7 sequenced cases	PTEN, BRAF, and ERBB3 mutations were each identified in one case	Not reported as a recurrent finding in the abstract
Mills et al., 2026 [3] Literature synthesis: extrauterine/ovarian MLA	KRAS alterations in 71/84 (84.5%) extrauterine/ovarian cases reviewed	PIK3CA 21/71 (29.6%); ARID1A 6/64 (9.4%); CTNNB1 5/69 (7.2%); PTEN 2/65 (3.1%)	1q gain in 35/39 (89.7%) evaluable extrauterine/ovarian cases

Note: Study cohorts are not directly comparable because sequencing platforms, case selection, and anatomic composition differ. The 2026 MLA Consortium synthesis is included to provide a current aggregate estimate; its denominators vary by gene according to data availability.

**Table 3:** Comparison of the present case with selected published studies of ovarian mesonephric-like adenocarcinoma.

Study [Ref.]	Cases/Setting	Recurrence/Metastatic Pattern	Diagnostic/IHC Features	Molecular Findings	Diagnostic Relevance	Comparison with Present Case
Koh et al., 2022 [43]	5 ovarian MLAs	One stage IA patient developed abdominopelvic peritoneal recurrence with liver and bilateral lung metastases 13 months after surgery.	Architectural diversity, small tubules and eosinophilic intraluminal secretions. All cases expressed ≥3 of GATA3, PAX2, TTF1 and CD10; WT1 negative; p53 wild-type.	Pathogenic KRAS mutations in all 4 tested tumors, including KRAS p.G12D in one case.	Shows that ovarian MLA may recur despite early-stage presentation and supports use of an integrated mesonephric IHC panel.	Shares the mesonephric-like IHC profile and KRAS p.G12D; recurrence sites differ from the bladder → vaginal cuff sequence in our case.
Euscher et al., 2023 [40]	33 extrauterine MLAs; ovary was the predominant extrauterine site	Extrauterine MLA showed a high propensity for recurrence, including distant recurrence.	79% were initially misclassified. GATA3 and/or TTF1 expression with reduced hormone-receptor expression facilitated recognition.	Among 20 tested tumors, KRAS was the most frequent alteration (15/20); TP53, SPOP and PIK3CA alterations were also reported.	Directly demonstrates the high risk of diagnostic misclassification of extrauterine MLA and the value of combined morphology and IHC.	Strongly parallels our case, in which different lesions received different initial diagnoses; our case adds urinary bladder mimicry of urothelial carcinoma.
Sugitani et al., 2024 [39]	1 ovarian MLA, FIGO IC1	Liver metastases developed 1 month after surgery; recurrent liver metastases were reported.	Tubular, glandular/papillary, cord-like and solid patterns; PAX8+, focal GATA3+, focal TTF1+, luminal CD10+; ER/PR/WT1 negative.	Molecular/phenotypic analysis was undertaken to identify therapeutic targets.	Illustrates rapid organ-specific metastatic recurrence and a characteristic integrated MLA phenotype.	Supports the metastatic potential of ovarian MLA; our case is distinctive for secondary bladder involvement mimicking a primary urinary tract tumor.
Ogawa et al., 2024 [10]	Institutional ovarian MLA series plus literature review (97 reported cases)	Two institutional cases experienced metastatic recurrence. In the literature review, recurrence was reported in 39% (26/67).	Study emphasizes recognition of ovarian MLA and its aggressive/recurrent behavior.	KRAS mutations were reported in 90% (38/42) of evaluable literature cases.	Provides broader evidence that recurrence is common and that KRAS alterations are frequent in ovarian MLA.	Shows that recurrence itself is not novel; the contribution of our case is the sequential ovary → bladder → vaginal cuff diagnostic course.
Mezzapesa et al., 2026 [1]	14 ovarian MLAs	57.1% experienced recurrence, predominantly as peritoneal metastases; median PFS 17.5 months.	Ovarian MLA alone or mixed with other histotypes; endometriosis was frequent.	KRAS mutations were present in all cases.	Recent series reinforces the aggressive behavior and high recurrence rate of ovarian MLA.	Contrasts with the unusual anatomic and diagnostic sequence in our case, particularly bladder involvement with urothelial mimicry.
Nakama et al., 2026 [56]	1 ovarian MLA	Ovarian MLA recurred in the lung as carcinosarcoma; liver metastases were also evaluated.	Metastatic tumor retained features supporting MLA origin; the report emphasizes TTF1, PAX8 and GATA3 and the importance of prior tumor history.	A KRAS mutation was demonstrated in metastatic disease.	Shows that recurrent MLA may undergo striking morphological evolution and mimic a different tumor type at a metastatic site.	Conceptually similar diagnostic pitfall: in our case, site-specific morphology/IHC led to urothelial and other gynecological diagnoses before MLA was recognized.
Present case	1 primary ovarian MLA with two subsequent anatomically distinct lesions	Extrinsic bladder wall involvement with preserved overlying urothelium, followed by vaginal cuff recurrence.	Primary: TTF1+, GATA3+, luminal CD10+, Napsin A+, ER−, p53 wild-type. Vaginal recurrence: PAX8+, TTF1+, GATA3+, CK7+, ER−, WT1−, CK20−, uroplakin III−, p53 wild-type. Bladder biopsy initially mimicked urothelial carcinoma.	KRAS p.G12D, PTEN and CTNNA1 alterations in the vaginal recurrence. Primary ovarian and bladder lesions were not molecularly tested; clonality therefore cannot be definitively established.	Demonstrates how MLA can be misclassified differently according to anatomic site and highlights the limitations of interpreting individual markers such as GATA3 or Napsin A in isolation.	Distinctive contribution: sequential diagnostic challenges across ovary → bladder → vaginal cuff, especially extrinsic bladder involvement mimicking primary urothelial carcinoma.

Abbreviations: MLA, mesonephric-like adenocarcinoma; IHC, immunohistochemistry; ER, estrogen receptor; PR, progesterone receptor; WT1, Wilms tumor 1; PFS, progression-free survival. Note: The table is intentionally selective and focuses on studies most relevant to recurrence, diagnostic mimicry, immunophenotype, and KRAS alterations. Molecular testing in the present case was performed only on the vaginal cuff recurrence; therefore, molecular clonality among the three lesions cannot be established.

## Data Availability

The original contributions presented in this study are included in the article. Further inquiries can be directed to the corresponding author.
